# Tuberculosis in Dialysis Patients in the Central Region of Morocco: What Is the Health-Care Delay?

**DOI:** 10.7759/cureus.30369

**Published:** 2022-10-16

**Authors:** Ghita El Bardai, Nadia Kabbali, Hanae Baba, Basmat Amal Chouhani, Tarik Sqalli Houssaini

**Affiliations:** 1 Nephrology, Dialysis, and Transplantation Department, Hassan II University Hospital, Fez, MAR; 2 Laboratory of Epidemiology and Health Science Research (ERESS), Faculty of Medicine-Fez, Sidi Mohammed Ben Abdelalh University, Fez, MAR

**Keywords:** difficult diagnosis, health care delay, peritoneal dialysis (pd), chronic hemodialysis, tb – tuberculosis

## Abstract

Introduction: Due to the predominantly cellular immunosuppression, infections are frequent in chronic dialysis patients, in particular tuberculosis (TB). The main objective of our study is to evaluate tuberculosis healthcare delay in dialysis patients and to raise the diagnostic challenge in these patients.

Material and methods: The study is retrospective and multicenter including tuberculosis cases of chronic dialysis patients either in hemodialysis (HD) or peritoneal dialysis (PD) in the central region of Morocco during a 10-year period between 2012 and 2021.

Results: We included 94 patients, five of whom were in PD, with a mean age of 50.79 ± 16.72 years, and a sex ratio of 0.67. The time between the initiation of dialysis and the onset of the clinical and biological presentation was 50.3 ± 67.12 months. The most frequent initial manifestations were an alteration of the general state (85.1%), a biological inflammatory syndrome (83%) or a prolonged fever (70.1%). Among our 94 patients, the diagnosis was confirmed with bacteriological evidence only in 18 cases (19.1%), by identification of Koch's Bacillus (BK) in 13 cases, by molecular biology test (GeneXpert; Cepheid, Inc., Sunnyvale, CA, USA) in five cases. The diagnosis of tuberculosis was presumptive in most cases (79 cases), i.e. 80.9%. Twenty-one patients underwent the interferon gamma release essay test (QuantiFERON; Qiagen, Hilden, Germany) which was positive in 14 patients. Thirty-four (36.1%) cases had a histological diagnosis. The remaining patients were offered a trial treatment. Tuberculosis localization was mostly extra-pulmonary (75.5%): lymph node (23.4%), pleural (13.8%), peritoneal (13.8%), whereas it was pulmonary in 23 cases (24.5%).

Most of our patients had a clear delay in management from symptom onset to initiation of anti-TB treatment 78.9% (time >21days) vs 21.1% (time ≤21days). The median time to management delay was 46.5 interquartile range (IQR) (28.5-90), the mean delay was 78.4 ± 87.9 (6-360). All patients were treated according to the RHZE/RH protocol (R: rifampicin, H: isoniazid Z: pyrazinamide and E: ethambutol), with a duration between six and 18 months. Side effects associated with anti-tuberculosis treatment were observed in half of the patients (51.1%). The evolution was favorable with remission and improvement of the general condition in 90% of cases. Two cases of resistance were noted in our series. The overall mortality was 7.7%.

Conclusion: We have confirmed a delay in the diagnosis and treatment of tuberculosis in chronic dialysis patients. This can be explained by the often atypical or incomplete clinical and paraclinical presentation and the extra-pulmonary localizations, making diagnosis difficult in this population whose prognosis remains poor. It is therefore necessary to establish a diagnostic approach that is adapted to the specificities of these high-risk patients within the framework of a national tuberculosis control program.

## Introduction

Tuberculosis (TB) remains a public health issue throughout the world and especially in developing countries such as Morocco, where it is still endemic. In 2019, the World Health Organization (WHO) estimated 35,000 new cases and 2,900 deaths related to tuberculosis in Morocco, hence a specific mortality rate of 8.1 per 100,000 inhabitants. In 2020, the number of registered cases including all forms of tuberculosis was 29,018 [1].

TB infection is more prevalent in patients on hemodialysis than in the general population [2]. This high frequency is due to many factors including deficits in cell and humoral-mediated immunity. In this patient category, the diagnosis is made difficult due to the atypical presentation, the increased frequency of extra-pulmonary localizations, and the negativity of bacteriological samples. Also, any delay in diagnosis will affect the prognosis of these patients.

There is no current consensus on the tuberculosis healthcare delay. Although there are studies of this delay in the general population [3,4], few studies have raised the issue for dialysis patients. It is therefore necessary to establish national standards that will allow for early diagnosis and timely treatment. This will improve the prognosis of these patients on the one hand and decrease the incidence of this pathology on the other. Our objective was to evaluate the tuberculosis healthcare delay in dialysis patients in the central region of Morocco while raising the clinical, paraclinical, and therapeutic particularities along with the difficulties of diagnosis in this population.

## Materials and methods

This is a retrospective and multicenter study including tuberculosis cases diagnosed according to the criteria of the National Tuberculosis Control Program [5] in chronic dialysis patients either on hemodialysis or on peritoneal dialysis in the central region of Morocco during a 10-year period between 2012 and 2021.

This region, also called the Fez-Meknes region, is one of the 12 new regions of Morocco established by the 2015 territorial division. The region includes two prefectures and seven provinces. It covers an area of 40,075 Km² with a population of 4,236,892 inhabitants according to the 2014 General Census of Population and Housing [6]. In this region, the number of public and private chronic hemodialysis centers has increased. In 2021, there were 51 hemodialysis centers (private and public) and only one peritoneal dialysis center at the Hassan II University Hospital installed in the region since 2009.

Definitions of tuberculosis cases

Definitions of Tuberculosis Cases (As per the Criteria of the National Tuberculosis Control Program [5])

A bacteriologically confirmed case: confirmed by the detection of Mycobacterium tuberculosis through molecular biology technique (Xprt MTB/RIF), direct examination of smears, and culture of pathological products.

A clinically diagnosed case (diagnosis without bacteriological evidence): based on clinical signs (deterioration of the general state, unexplained fever, cough, etc.) persistent beyond two weeks, radiological abnormalities, positivity of tuberculin test and interferon gamma release assay, histology presence of specific lesions (necrotizing granulomatous inflammation composed of central necrotic zone surrounded by epithelioid histiocytes), and predisposition (positive history of contact with TB, immunocompromised subjects).

Definition of Deadlines

Tuberculosis health-care delay: time interval between the onset of symptoms and the start of anti-tuberculosis treatment.

Delay in care if the time between diagnosis and treatment exceeds 21 days according to WHO recommendations [4,7].

Data collection

Data were collected through a data collection form completed using patient charts. The data collected included demographic data (sex, age, socio-economic status, etc.), history (diabetes, hypertension, date of initiation of dialysis, tuberculosis infection, etc.), clinical presentation (alteration of general condition, weight loss, unexplained prolonged fever, chills, night sweats, respiratory manifestations, ascites, adenopathy), time of onset of tuberculosis in relation to the beginning of dialysis, laboratory findings consistent with an inflammatory syndrome (white blood cell count, C reactive protein (CRP), ferritin), microbiological examinations (culture Koch bacillus), molecular biology test performed on a blood draw, pleural effusion, peritoneal effusion, interferon gamma release essay (QuantiFERON, Qiagen, Hilden, Germany), radiological data (Chest X-ray and/or CT scan, abdominal ultrasound and/or CT scan), pathology results of the performed biopsies, the protocol and duration of the anti-tuberculosis treatment, the drugs used and the adaptation of dosage made according to ICAR [8], the associated side effects, and the evolution of the patients including recovery, relapse, resistance to treatment, and death.

Statistical analysis

The data were entered into an Excel sheet (Microsoft, Redmond, WA, USA) and analyzed using SPSS software package V20 (IBM Corp., Armonk, NY, USA). In the descriptive part of the analysis, quantitative variables were expressed as mean ± standard deviation and/or median with interquartile range (IQR), and qualitative variables as percentages. In order to determine factors related to tuberculosis management delay among sample patients, the comparison between means was performed using the student test when normal distribution in each comparison group was shown; if not, Mann-Whitney non-parametric test was used. The comparison of proportions was performed using the Chi-square test. The level of significance adopted was p < 0.05.

## Results

The number of patients on chronic dialysis increased during the study period in the central region. In 2021, there were 3941 patients on hemodialysis and 53 patients on peritoneal dialysis with a dialysis prevalence of 826 patients per million inhabitants per year (according to data from the Moroccan Society of Nephrology). We collected 94 dialysis patients with tuberculosis during the study period that were distributed in different chronic hemodialysis centers in the central region with an incidence of 6.8 per thousand chronic dialysis patients per year in 2021. The sex ratio was 0.67 with 56 women and 38 men. The average age was 50.79 ± 16.72 years (12-88 years); most of them were of an average (56.4%) or low (40.4%) socio-economic level. Of the 94 patients, five were on peritoneal dialysis. History was positive for tuberculosis contage in 13 patients (13.8%), while eight patients (8.5%) had a history of tuberculosis before starting dialysis, and diabetes was found in 13 patients (13.8%). The cause of end-stage renal disease was undetermined in 51.4% of cases; diabetes-related kidney disease was found in 11% of patients. Seniority in dialysis was seven years ± 4.81 (1-22). The average time between the initiation of dialysis and the onset of clinical and biological symptoms was 50.3 months ± 67.12 (0.5-450 months) (Table 1).

**Table 1:** Demographic and clinical characteristics of the patients (n = 94).

Demographic and clinical characteristics of the patients (n = 94)	
Male n (%)	38 (40.4)
Female n (%)	56 (59.6)
Age (years) Mean ± SD (Range)	0.79 ± 16.72 (12–88)
Low socioeconomic level n (%)	38 (40.4)
Medium socioeconomic level n (%)	53 (56.4)
High socioeconomic level n (%)	3 (3.2)
Tuberculosis contagion history n (%)	13 (13.8)
Tuberculosis history n (%)	8 (8.5)
Diabetes history n (%)	13 (13.8)
Hemodialysis n (%)	89 (94.7)
Peritoneal dialysis n (%)	5 (5.3)
Duration of dialysis (years) Mean ± SD (Range)	7 ± 4.81 (1–22)
Delay between the initiation of dialysis and the onset of clinical and biological symptoms (months) Mean ± SD (Range)	50.3 ± 67.12 (0.5–450)

The most frequent clinical signs included deterioration in general condition with weight loss in 85.1% of cases, unexplained prolonged fever in 70.1% of cases, and typical respiratory manifestations (cough, hemoptysis, chest pain, and dyspnea) in 46.8% of cases with cough as the main symptom. Physical examination revealed the presence of pleural effusion syndrome in 25.5% of cases, ascites in 16% of cases, and peripheral lymphadenopathy in 23.4% of cases mostly of a cervical localization (Table 2).

**Table 2:** Clinical and paraclinical manifestations of tuberculosis (n = 94).

	n (%)
Fever/pyrexia of unknown origin	66 (70.2)
Loss of body weight and condition	80 (85.1)
Respiratory manifestations	44 (46.8)
Pleural effusion	24 (25.5)
Ascities/peritonitis	15 (16)
Lymphnodes enlargement	22 (23.4)
Abnormal chest radiograph	50 (53.2)
Positive Acid fast bacilli sputum	13 (13.8)

Laboratory results demonstrated an inflammatory syndrome reported in 83% of patients with high CRP levels of 99.7 mg/l ± 92.7 (1-578) on average. All patients underwent a Chest X-ray and were tested for Koch's bacillus (KB) in the sputum. Radiological abnormalities were present in 50.3% cases and the search for KB in sputum was positive only in 13.8% cases. The major radiological images found were pleural effusion in 22 cases and unsystematized and unexcavated lung opacities in 12 cases, while the most suggestive image (excavated opacity) was found in only two patients. CT abnormalities pointing to an extra-pulmonary localization were present in 19% of cases (bone involvement: 7.69%, lymph node involvement: 5.7%, multifocal involvement: 5.7%), and pulmonary in 13.4% cases. It should be noted that the cerebro-thoraco-abdomino-pelvic CT was normal in 10 patients.

Tuberculosis was confirmed with bacteriological evidence in only 18 cases, which is 19.1%, in addition to the 13 patients who tested positive for KB, and 23 patients underwent a GeneXpert test (Cepheid, Inc., Sunnyvale, CA, USA) wherein five were positive. The diagnosis of tuberculosis was a presumptive one in most cases (79 cases), i.e., 80.9%. Of the 21 patients that underwent a Quantiferon test, 14 patients were positive. Thirty-four (36.1%) cases underwent a histological diagnosis (lymph node biopsy: n=15, pleural biopsy: n=8, peritoneal biopsy: n=7, bone biopsy: n=3, digestive biopsy: n=1). The rest of the patients received trial treatment. The tuberculosis localization was most often extrapulmonary (75.5%): lymph node (23.4%), pleural (13.8%), peritoneal (13.8%), and pulmonary in 23 cases (24.5%) including three cases of miliary (Table 3).

**Table 3:** Site of tuberculosis in dialysis patients (n = 94).

Name of site	n (%)
Pulmonary	23(24.5)
Pleural	13(13.8)
Lymphnode	22(23.4)
Peritoneal	13(13.8)
Bone	11(11.7)
Disseminated	8(8.5)
Pericardial	3(3.1)
Digestive	1(1.1)

**Figure 1:**
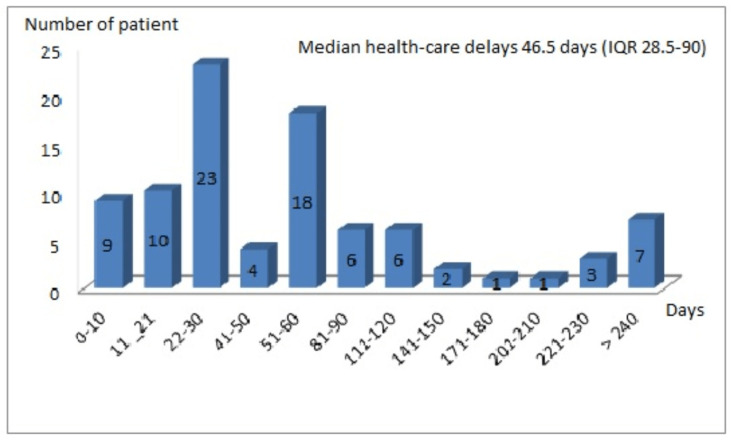
Distribution of estimated healthcare delays IQR: interquartile range

Most of our patients experienced a clear delay in care between the onset of symptoms and the start of anti-tuberculosis treatment: 78.9% (time > 21d) against 21.1% (time ≤ 21d). The median time to treatment was 46.5 IQR (28.5-90), the mean time was 78.4 ± 87.9 (6-360) (Figure 1, Table 4). In case of univariate analysis, none of the factors studied related to the delay in treatment (age, sex, socio-economic level, ATCD, number of symptoms, radiological abnormalities, KB positivity, dialysis modality, case definitions, location of tuberculosis, remission, death) were statistically significant.

**Table 4:** Median health-care delays by demographic, clinical, paraclinical factors.

Characteristics	Patients n (%)	Health-care delay, days (IQR)
All patients	94 (100%)	46.5 (28.5–90)
Age ≤ 65 years	78 (83)	45 (30–90)
Age > 65 years	16 (17)	60 (14–60)
Male	38 (40.4)	37.50 (20.75–97.50)
Female	56 (59.6)	60 (30–90)
Diabetic	13 (13.8)	13.50 (30–195)
Not diabetic	81 (86.2)	60 (30–90)
Low socioeconomic level	38 (40.4)	60 (30–120)
Medium and High socioeconomic level	56 (59.6)	45 (22.5–75)
Number of symptoms ≤ 2	39 (41.5)	48 (21.5–75)
Number of symptoms > 2	55 (58.5)	45 (30–120)
Abnormalities Chest X-ray	50 (53.2)	21 (30–82.5)
Normal Chest X-ray	44 (46.8)	30 (60–120)
Positive Acid fast bacilli sputum	13 (13.8)	30 (10–60)
Negative Acid fast bacilli sputum	81 (86.2)	60 (30–90)
Hemodialysis	89 (94.7)	48 (23.5–90)
Peritoneal dialysis	5 (5.3)	30 (30–120)
Confirmed diagnosis	18 (19.1)	30 (24.75–45)
Presumptive diagnosis	79 (80.9)	28.5 (60–97.50)
Deaths	7 (7.7)	45 (30–90)
No deaths	87 (89)	30 (60–210)

All patients were treated using a therapeutic regimen of rifampicin, isoniazid, pyrazinamide, and ethambutol (RHZE (attack phase)/RH (maintenance phase)) with the duration of treatment between six and 18 months. Side effects associated with anti-tuberculosis treatment were observed in half of the patients (51.1%) and the most frequent ones were digestive (34%), mostly vomiting, abdominal pain, and diarrhea. Neurological side effects were observed in 20.2% of cases with predominance of neuropathies requiring the introduction of vitamin therapy that improved clinically without the need to discontinue the treatment. The outcome was favorable with remission and improvement in general condition in 90% of cases. We noted two cases of resistance in our series, and four patients presented a relapse during the study period. The overall mortality was 7.7%.

The five patients on peritoneal dialysis experienced peritoneal tuberculosis resistant to the usual antibiotics. Four patients underwent a positive moleculer test on a peritoneal fluid sample. The median time to diagnosis and treatment was 30 days IQR (30-120). The evolution was marked by remission in the five patients. All patients were switched to hemodialysis with catheter removal without any death cases.

## Discussion

Tuberculosis remains one of the deadliest, most infectious, and most contagious diseases and is currently among the top 10 causes of death worldwide [9]. Its frequency in dialysis is extremely variable in literature. In a literature review, the authors note a relative risk of 6.9 to 52.5% [10]. However, this risk remains 7 to 250 times higher than in the general population [11,12]. In our study, we also note an incidence of tuberculosis in dialysis patients eight times higher than in the general population, i.e., 6.8 per thousand chronic dialysis patients per year compared to 79 cases per 100,000 inhabitants per year in the central region of Morocco in 2021 [1].

This high frequency is because of factors specific to chronic dialysis patients, notably cellular immune deficiency, the chronic inflammatory state induced and maintained by the bio-incompatibility of the dialysis material, undernutrition, and high ferritin levels [2,11,13], which are associated with other factors found in the general population (age, low socioeconomic level, promiscuity, lack of vaccination, recent tuberculosis contagion, toxic habits, immunosuppressive treatment, and comorbidities) [14].

The diagnosis of tuberculosis remains a huge challenge due to the atypical clinical presentation, the increased frequency of extra-pulmonary localizations, and the negativity of bacteriological samples [2,11,12,15-17]. In our series, the most frequent manifestations included an alteration of the general state (83%) and a prolonged fever (66%), which was in line with the data in the literature [18]. It should be noted that tuberculosis can present as a prolonged fever of unknown origin in 43.3% to 77.4% of patients in endemic areas [19]. Therefore, prolonged fever in dialysis patients should routinely raise the possibility of tuberculosis.

In our study, the diagnosis was confirmed using bacteriological evidence in only 19.1% of patients. According to the literature, skin tests are not very sensitive and are almost always negative. It reflects the state of anergy secondary to the decline in cell-mediated immunity [20]. Chest radiography is not very helpful because of the high frequency of extrapulmonary localizations and the variable and, often, nonspecific semiology. Polymorphic or interstitial infiltrate is the most common, accounting for 30% of cases, followed by pleural effusion in 27% of cases. In 18% of cases, no abnormality is found [12]. In our series, radiological abnormalities were present in 50.3% of cases with pleurisy as the main lesion in 46% of cases. The use of CT in our series increased the diagnostic presumption, especially in extra-pulmonary types. Bacteriological evidence is rarely obtained in either pulmonary or extrapulmonary localization. The detection of KB in dialysis patients with tuberculosis varies from 19.2% to 42%. In our study, KB was detected in only 13.8% of patients. An anatomopathological examination is generally essential to guide the diagnosis; in our series, the biopsy was positive in 36.1% cases with a variation in the histological diagnosis of tuberculosis in the literature from 22.7% to 67.1% [11]. The extra-pulmonary localization of tuberculosis is the most frequent in dialysis patients. It is characterized in this population by evolving quietly, representing between 51.6% and 100% of localizations according to the series [2,11,15,17,21,22], which is similar to our results of 75.5%.

These clinical and paraclinical manifestations are not very specific, which makes the diagnosis of tuberculosis in dialysis patients difficult. They can be attributed to inadequate dialysis, fluid overload, uremic symptoms, or a complication of hemodialysis, which may participate in a diagnostic and therapeutic delay and ultimately worsen the prognosis of patients [17].

There is no current consensus on the tuberculosis healthcare delay. While there are studies of this delay in the general population [3,4], few studies have raised the issue in dialysis patients. Our study from an endemic country is among the first studies to evaluate the time to diagnosis and treatment of tuberculosis in dialysis patients. The median delay in days found in this population is 46.5 IQR (28.5-90) with a mean delay of 78.4 days ± 87.9 (6-360), which greatly exceeds the recommended level by the World Health Organization, which suggests a management delay of 21 days in the general population [4,7]. In the Tunisian study, the mean time to care for dialysis patients with tuberculosis was 113.52 days (30-360) [15]. Our delay also exceeds the delays reported in the literature for the general population; a median health-care delay of 24 days (IQR 10-45) was found in a recent American study [4]. The 2006 WHO study in countries with high prevalence of tuberculosis noted a median delay between symptom onset and treatment ranging from 46 days in Iraq to 127 days in the Islamic Republic of Iran [3]. These studies have demonstrated several significant factors related to the delay in diagnosis and treatment, including age, sex, socioeconomic status, past medical history, number of symptoms, radiological abnormalities, KB positivity, location of tuberculosis, and factors related to the health care system of each country [3,4]. In our study sample, four-fifths of the patients experienced a delay in management (delay > 21 days), so the remaining patients who do not undergo a delay (≤ 21 days) constitute only one-fifth of the sample (about 19 patients). With these numbers of patients, it is difficult to perform comparison tests with the required power to identify possible cofactors related to delay in management, including those discussed in the studies cited above.

The average time between the beginning of dialysis and the detection of tuberculosis is variable, ranging from 4 to twenty-six months. In general, tuberculosis seems to occur relatively early with respect to the start of hemodialysis in relation to an enhanced decrease in cell-mediated immunity favoring the activation of old foci, so it is more often a reactivation of latent tuberculosis than a new contamination [12,15]. In our series, half of the patients had reported their disease during the first two years of dialysis. The pharmacology of anti-tuberculosis drugs is significantly altered in dialysis patients. Proper handling of these drugs is necessary to ensure the efficacy of the treatment while avoiding the frequently described neuropsychiatric, hepatic, and gastrointestinal side effects. The reported frequency of these side effects is between 46.3% and 56% [15,23].

Higher mortality was reported in early series and more recent studies show improvement [2]. The high mortality rate has been attributed to delayed diagnosis and spread of tuberculosis [12,15]. In our series, the mortality rate was about 7.7%, which was lower when compared with other studies wherein the rates were 36.58% and 50% in the Tunisian and Indian groups, respectively [11,15].

In the end, we suggest routine screening for latent tuberculosis in dialysis patients using an interferon-gamma release test (QuantiFERON-TB Gold), which has been shown to be sensitive and inexpensive. Several studies have demonstrated the cost-effectiveness of screening for this infectious complication in this type of high-risk population. We also recommend adapting the diagnostic criteria or case definition for tuberculosis in chronic dialysis patients. Negativity of the usual tests cannot rule out tuberculosis when suggestive history, symptoms, or clinical signs are present. The high suspicion of tuberculosis in dialysis patients should always be kept in mind, especially in endemic areas. Repeated investigations, including molecular biology testing, should also be considered for diagnosis.

## Conclusions

We have confirmed the delay in diagnosis and treatment of tuberculosis in chronic dialysis patients. This can be explained by the often atypical or incomplete clinical and paraclinical presentation and the extra-pulmonary localizations, thereby making the diagnosis difficult in this population whose prognosis remains poor. More extensive studies are required to determine the factors related to this delay in order to establish a diagnostic approach tailored to the specificities of this high-risk population within the framework of a national tuberculosis control program so as to improve the morbidity and mortality of these patients and control the incidence of tuberculosis in general.
